# Field Efficacy, Sub-lethal, and Biochemical Effects of Certain Biorational Insecticides Against the New Intruder, *Spodoptera frugiperda* in Bani-Suef, Upper Egypt

**DOI:** 10.1007/s13744-023-01064-y

**Published:** 2023-07-25

**Authors:** Wael M. Khamis, Ahmed M. El-Sabrout, Rima Shahin, Elham F. Abdel-Rahim

**Affiliations:** 1 Plant Protection Research Institute, Agricultural Research Center, Al-Sabhia, Alexandria, Egypt; 2 Department of Applied Entomology and Zoology, Faculty of Agriculture (El-Shatby), Alexandria University, Alexandria, 21545 Egypt; 3 Plant Protection Research Institute, Agricultural Research Center, Sides Agriculture Research Station, Al-Giza, Egypt

**Keywords:** Fall armyworm, *Beauveria bassiana*, Spinetoram, Lipase, Glutathione-S-transferase

## Abstract

Frequent inspections on sorghum and maize crops during seasons of 2021 and 2022 in some regions in Bani-Suef governorate, Egypt, discovered unprecedented invasions of *Spodoptera frugiperda* (J. E. Smith). Accordingly, our study on *Beauveria bassiana* and spinetoram was supporter to the Food and Agriculture Organization’s tendency in adopting biorational insecticides against *S. frugiperda* in Egypt*.* Exposure toxicity of LC_25_ values at 48 h of *B. bassiana* were 2.7 × 10^6^ and 5.2 × 10^6^ conidia mL^−1^ and spinetoram were 0.019 and 0.048 mg L^−1^ against the 2nd and 4th instar larvae laboratory strain of *S. frugiperda*, respectively. Sub-lethal effects (LC_25_) were accomplished on biological parameters against both instar larvae. LC_25_ of *B. bassiana* reduced adult emergency (89.91 and 91.05%) more than spinetoram (75.99 and 79.49%) against the 2nd and 4th instar larvae, respectively. The 2nd instar larvae exposed to LC_25_ of *B. bassiana* suppressed female fecundity (0.00 eggs) more than spinetoram (19.74 eggs). Enzymatic activity of lipase in hemolymph, fat bodies, and mid-gut of the 4th instars at 48 h showed significant drop in *B. bassiana* more than spinetoram. Glutathione-S-transferase (GST) levels in hemolymph for both insecticides were equal and exceeded the control. Fat bodies and mid-gut possessed the highest GST activity in *B. bassiana* followed by spinetoram and the control. Residual efficacy of spinetoram exceled *B. bassiana* at their field rates under semi-field condition in Bani-Suef along the two seasons of maize crop against both instars. Eventually, *B. bassiana* alongside spinetoram could afford good control especially on early instar larvae of *S. frugiperda*.

**Supplementary Information:**

The online version contains supplementary material available at 10.1007/s13744-023-01064-y.

## Introduction

The fall armyworm, *Spodoptera frugiperda* (J. E. Smith) (Lepidoptera: Noctuidae), is one of the major invasive polyphagous pests that habitat more than 353 plant species, especially maize, sorghum, sugarcane, turfgrass, cotton, and vegetable crops (Montezano 2018; Gamil 2020; Timilsena et al. 2022). Since 2016, *S. frugiperda* has spread rapidly over 44 countries in West Africa (Day et al. 2018; Kassie et al. 2020). In Egypt, *S. frugiperda* was first recorded on maize fields in 2019 in Komombo, Aswan Governorate (Food and Agriculture Organization (FAO) 2019; Dahi et al. 2020, Gamil 2020). Thereafter, it overspread Upper Egypt governorate towards the north on maize and sorghum crops in 2021 (Mohamed et al. 2022). It caused enormous damage in Africa on sorghum (Hailu et al. 2021) and maize crops, with an annual loss in yield up to 17.7 million tons (Day et al. 2018; International Plan Biotechnology Outreach (VIB) 2019; Kassie et al. 2020).

In this respect, endeavors throughout three years (2020–2022) of global action by *S. frugiperda* monitoring and early warning system (FAMEWS) was settled to curb the threat of *S. frugiperda* to new areas of Africa and Asia. Since the *S. frugiperda* might possess a quick resistance to many active substances, science-based biotechnology solutions, predominantly biological control, bio-pesticides applications, and less toxic chemicals should be considered in new infested areas (Food and Agriculture Organization (FAO) (2020)).

Entomopathogenic fungi (EPF), *Beauveria bassiana* (Bals.) Vuillemin (Hypocreales: Cordycipitaceae) potentially exploits as colonized endophyte belongs to class deuteromycete. *Beauveria bassiana* can released secondary metabolite mycotoxin called beauvericin (cyclic hexadepsipeptides) in their host plants and subsequently cause a white muscardine toxic disease to herbivorous insects (Shah and Pell 2003; Mwamburi 2021). On the other hand, spinetoram is a fermented product of *Saccharopolyspora spinosa* in the form of a multicomponent tetracyclic macrolide. Spinetoram belongs to class of spinosyns who acts as nicotinic acetylcholine receptor allosteric modulators-site 1 besides gamma amino butyric acid-gated chloride channels blockers (Insecticides Resistance Action Committee (IRAC) 2020; Environmental Protection Agency (EPA) 2009).

Indeed, pathogenesis and metabolic activities of EPF, *B. bassiana* mainly are potential source of lipases, which confer a potent virulence factor for *B. bassiana* (Vici et al. 2015). Lipases involve the process of hydrolyses on ester bonds of lipoproteins, fats, and waxes in the interior integument of the insect and subsequently involve the cuticle adhesion and penetration (Ali et al. 2009; Supakdamrongkul et al. 2010; Silva et al. 2010; Dhawan and Joshi 2017). In addition, glutathione-S-transferase (GST) possessed a vital role in defending insects that may be exposed to oxidative stress via detoxification and cellular antioxidant processes led to conjugate the yield of reduced glutathione to the electrophilic sites of EPF (Lumjuan et al. 2005; Li et al. 2007; Wu et al. 2020). GST activity increased in *Spodoptera litura* (Fabricus) (Lepidoptera: Noctuidae) as a response to secondary metabolite mycotoxins produced by *Beauveria brongniartii* (Sinha et al. 2016; Wu et al. 2020). In fact, the activity of detoxification enzyme, GST significantly increased in the larvae of *Spodoptera littoralis* (Boisd.) (Lepidoptera: Noctuidae) when exposed to spinetoram more than in the control (Ismail 2020). Recent studies showed effectiveness of *B. bassiana* as biological control against the early instar of *S. frugiperda* in laboratory (Shahzad et al. 2021) and field (Ramos et al. 2020). Meanwhile, spinetoram was exploited in controlling leaf miners, thrips, and lepidopteran’s larvae (EPA 2009). In Gowa, Indonesia, the field trials carried out on spinetoram against *S. frugiperda* showed a significant increase in maize yield from 5.2 to 10.7 tons per hectare in treated areas (Nonci et al. 2020). In this regard, our study work first performed frequent inspections for the new potential invasion of *S. frugiperda* on main hosting crops in some regions of Bani-Suef governorate, Upper Egypt. Secondly, comparative studies of sub-lethal concentrations of *B. bassiana* and spinetoram on biological parameters and enzymatic aspects were carried out in the laboratory on early and mid-instar larvae of *S. frugiperda*. Finally, the residual toxic effects of these selected biorational insecticides against the early and mid-instar larvae of *S. frugiperda* were investigated under semi-field conditions in Bani-Suef governorate. Thence, our study followed in the footstep of FAMEWS in focusing on the evaluation of biorational insecticides, *B. bassiana* and spinetoram against *S. frugiperda* in new infested areas in Egypt, to curb the probable resistance that may arise due to the overuse and unwise applications of conventional insecticides.

## Material and methods

### Field survey of *Spodoptera frugiperda* infestation

Frequent inspections on *S. frugiperda* infestation were carried out during May and November in seasons of 2021 and 2022 in some field locations of Bani-Suef governorate Upper Egypt. These inspections were accomplished on maize, *Zea mays* (Giza 2, Sids 128 and hybrid 2031) and *Sorghum bicolor* (L.) (various hybrids under evaluation of ARS and Baladi variety). The field survey compromised of four villages that exclusively distributed at Ezbit Al-Hakem (28°54′29.4″N: 30°55′21.9″E) and ARS, Sids (28°54′29.5″N: 30°57′01.1″E) in Biba region, as well as Bani-Hallah (28^o^54′23.7″N: 30^o^55′08.7″E) and Ghaftan (28°57′05.5″N: 30°49′10.3″E) in Sumasta region. The routine inspections were weekly implemented along the period of survey. Each routine inspection assigned about 40 samples of plants for each crop by randomly selections in the foregoing locations for each region in Bani-Suef governorate.

### Rearing conditions of *Spodoptera frugiperda*

Rearing of *S. frugiperda* was accomplished on fresh castor leaves, *Ricinus communis* (L.) under the laboratory conditions (27 ± 2 °C, RH 60 ± 5%) according to El-Defrawi et al. (1964) and El-Sabrout (2009). The collected colonies of *S. frugiperda* field strain from different regions of Bani-Suef governorate were reared in laboratory of *S. frugiperda* up to the 5th generation, in which the 2nd and 4th instar larvae were submitted to the evaluation of biological aspects and enzymatic activity, whereas semi-field trials were conducted on both instar larvae of *S. frugiperda* that nutritionally adapted on fluffy young leaves of maize for two generations under the same laboratory conditions.

### Tested insecticides

*Beauveria bassiana* (Biosect [32 × 10^6^ colony-forming unit (CFU) mg^−1^] WP classified as entomopathogenic fungi) was applied with the field rate of 400 gm 200 L^−1^ water fadan^−1^. Spinetoram (Radiant® 12% SC belongs to spinosyns) was applied with the field rate of 50 mL 200 L^−1^ water fadan^−1^.

#### Toxicity studies

The toxicity test of *B. bassiana* and spinetoram were carried out against each of the 2nd and 4th instar larvae of *S. frugiperda* at 48 h of exposure on castor bean leaves in order to estimate their sub-lethal values at LC_25_. Thereby, these LC_25_ values were tested on the biological aspects of both tested instars at 48 h of exposure on castor bean leaves. Meanwhile, the enzymatic activity was tested only on the 4th instars. Ultimately, the semi-field experiments were conducted on *Zea mays* crop against both instar larvae from a group that previously adapted to feed on maize leaves for 2 generations in the laboratory.

#### Lethal concentration curve

Toxicity of *B. bassiana* and spinetoram were carried out against the 2nd and 4th instar larvae of *S. frugiperda* at 48 h of exposure using dipping technique of El-defrawi et al. (1964) by castor bean leaf disks. Seven concentrations were assigned for the tested insecticides except eight concentrations for spinetoram on the 4th instar larvae as explained by the term, degree of freedom (*df*) in Table 1. The concentration ranges of *B. bassiana* were 0.3 × 10^7^ to 3 × 10^9^ and 0.7 × 10^6^ to 5.8 × 10^7^ conidia mL^−1^, while spinetoram were 0.35 × 10^−2^ to 0.50 and 0.2 to 2.00 mg L^−1^ against the 2nd and 4th instar larvae, respectively. Leaf disks were dipped in each concentration for 20 s and then get out for dryness at temperature of 27 ± 2 °C. Three replicates of glass cups (250 cm^3^) were assigned for each tested concentration. Equal numbers of treated leaf disks at each concentration (enough to nourish the tested larvae over 48 h) were distributed for each replicate. Ten of either 2nd or 4th instar larvae (24 h of pre-starvation) were inserted to each replicate. Mortality percentages were calculated and corrected versus to the control treatment according to the equation of Abbott (1925) and then submitted to probit analysis (Finney 1971). The LC_25_ value was determined for each tested insecticide. Numbers of conidial cells corresponding to the LC_25_ of *B. bassiana* were estimated in suspension at concentration of 1 mg mL^−1^ of autoclaved deionized water on Neubauer hemocytometer (Moore 2018).

#### Sub-lethal effects

Biological aspects on the 2nd or 4th instar larvae of *S. frugiperda* post-treatment with LC_25_ values of *B. bassiana* and spinetoram at 48 h of exposure were carried out in ARS, Sids, Bani-Suef. Castor bean leaves were immersed in the LC_25_ values of each tested insecticide and distilled water for the control, then after it left to dry. Each treatment was replicated three times. In each replicate, 100 of identical larvae of either 2nd or 4th instars were reared and feed in glass container (1000 cm^3^) on the treated castor bean leaves. After 48 h of exposure, survival larvae were picked up and transferred to another uncontaminated container with untreated leaves. Then, the survived larvae in all treatments were monitored for their biological aspects. The biological parameters included percentage of pupation, pupal duration, percentage of adult emergence, adult fecundity rate, and percentage of eggs hatching

#### Enzymatic activity

##### Crude extract preparation

The 4th instar larvae of *S. frugiperda* were treated with the LC_25_ values of the tested insecticides at 48 h of exposure in compare to the control. Three replicates were assigned for each treatment. Each replicate contained adequate portion of treated castor leaves that introduced to ten pre-starved larvae for 24 h. Thereafter, the survived larvae of each treatment were prepared for biochemical examination in the Laboratory of insect physiology, Faculty of Agriculture (El-Shatby), Alexandria University. Hemolymph was taken from a severed proleg and placed in Eppendorf tubes with numerous crystals of phenylthiourea, to prevent melanization. The enzyme activity was determined in the cell-free plasma fraction after centrifugation at 10,000 × *g* for 15 min at 4 °C (human centrifuge, TGL-16XYJ-2, 16,000 rpm, Korea). The luminal content and fat bodies were removed, and the tissues were rinsed in a pre-cooled saline solution (1 M NaCl). Tissues were homogenized in a 1:2 (w/v) ratio of specified enzyme buffers (20 mM Tris–HCL, pH 7.4 containing 0.25 M sucrose) for lipase assay and (100 mM potassium phosphate, 2 mM EDTA, pH 7.0 containing 0.25 M sucrose) for GSTs assay, using a homogenizer disperser Ultra Turrax (IKA-Werke, Staufen, Germany) on ice bath. The homogenates were centrifuged for 10 min at 10,000 × *g* at 4 °C, with the pellet discarded and the supernatant used as an enzyme source.

##### Lipase activity assay

Lipase activity in prepared samples was measured using the lipase quantitative kinetic assay kit (Ben Biochemical Enterprise, Italy) (LIP3542), according to the manufacturer’s protocol and spectrophotometer (UNICO, SP2100 UV, China) at 575 nm.

##### Glutathione-S-transferase activity assay

The cytosolic glutathione-S-transferase (GST) was obtained by centrifuging the supernatant at 100,000 g for 60 min at 4 °C. The GST activity was measured spectro-photometrically using a kit (Bio-diagnostic Research reagents, CAT. NO. GT 25 19), samples were read at 340 nm (DU-8200, UV/Vis Spectrophotometer, China), as directed by the manufacturer.

### Residual toxicity

Semi-field experiments were conducted during seasons of *Zea mays* crop (variety of Sids 128) with in the early July in 2021 and mid of June in 2022 against the 2nd and 4th instar larvae of *S. frugiperda* at ARS, Sids, Bani-Suef governorate. The maize crop plantations disciplined the guidelines of crop management practices (Martin et al. 2015). Semi-field experiments were submitted to a randomized complete block design. Each treatment had three replicates of plot (40 m^2^). Foliar sprays for each insecticide treatment were conducted by Knapsack sprayer equipment (CP3) at the rate of 200 L fadan^−1^. Each insecticide was sprayed separately according to their recommended field rate (FR) dosages and with water in the control. Samples of adequate mid-aged leaves were collected from treated and untreated (control) plots in perforated bags. These samples were collected at 0, 3, 5, 7, and 10 days after treatment (DAT). Collected samples were delivered timely to the laboratory to resume the toxicity test on the 2nd and 4th instar larvae under conditions of 27 ± 2 °C, RH 60 ± 5%. Equal portions of leaf sample were introduced to nourish ten larvae of either 2nd or 4th instars in a glass bottle (250 cm^3^). Each treatment contained three replicates of glass bottle. Mortalities after 48 h of exposure and long-termed toxicities at 0, 3, 5, 7, and 10 DAT were recorded and corrected according to the equation of Abbott (1925).

### Statistical analysis

All the obtained results of laboratory studies and semi-field trials were submitted to analysis of variance (one-way ANOVA). Means were determined for significance at LSD 0.05 test by using software of Statistical Analysis System (SAS) Institute (2002).

## Results

### Toxicity of the tested insecticides

The value of LC_25_ of *B. bassiana* against the 2nd instar larvae (2.7 × 10^6^ conidia mL^−1^) was significantly lower than the 4th instar larvae (5.2 × 10^6^ conidia mL^−1^) at 48 h of exposures. Meanwhile, the value of LC_25_ of spinetoram against the 2nd instar larvae (0.019 mg L^−1^) was significantly lower than the 4th instar larvae (0.048 mg L^−1^) at 48 h of exposures. In each instar larvae, the toxicity of spinetoram was shown to be much higher than that of *B. bassiana* (Table 1).

**Table 1:** Sub-lethal concentrations (LC_25_) of the selected insecticides against the 2nd and 4th instar larvae of *Spodoptera frugiperda* at 48 h of exposure

Insecticides	Instars	LC_25_ (95% CL)^1^	Slope ± SE^2^	*χ*^2^	*df*
*Beauveria bassiana* (32 × 10^6^ CFU mg^−1^ WP)	2nd	2.7 × 10^6^ (2.2 × 10^6^–3.3 × 10^6^)	1.13 ± 0.67	10.66	5
	4th	5.2 × 10^6^ (4.1 × 10^6^–6.5 × 10^6^)	1.22 ± 0.61	5.39	5
Spinetoram (12% SC)	2nd	0.019 (0.015–0.025)	1.30 ± 0.22	3.39	5
	4th	0.048 (0.029–0.080)	1.40 ± 0.56	4.48	6

^1^Concentration assigns to kill 25% of the larvae with (95% confidence limits) expressed by conidia mL^−1^ for *B. bassiana* and mg L^−1^ for spinetoram

^2^Standard error

### Sub-lethal effects

The obtained data of biological aspects on the effects of *B. bassiana* and spinetoram at their LC_25_ values on the 2nd instar larvae showed equipollent prolongations on the pupation percentages and pupal duration; meanwhile, the 4th instars had equivalent prolongations on the pupal duration. Moreover, no significant differences were found between the selected insecticides and their control on adult longevity (Tables 2 and 3). Regarding to the data of the 2nd instar larvae of *S. frugiperda* (Table 2), significant decreases on the adult emergency were mostly prevailed in the treatment of spinetoram at LC_25_ (75.99%) over *B. bassiana* at LC_25_ (89.91%) compared to the control (95.50%). Contrary, significant obstruction on the adult moth female to lay eggs (fecundity) was fulfilled by LC_25_ of *B. bassiana* (0.00 eggs), which was higher than LC_25_ of spinetoram (19.74 eggs) in compare to the control (797.90 eggs). Therefore, the 2nd instar larvae exposed to LC_25_ of *B. bassiana* were unable to survive for their life cycle completion. Furthermore, LC_25_ of spinetoram significantly decreased the egg hatchability (16.42%) more than the control (72.51%).

**Table 2:** Effect of tested insecticides at their LC_25_ values on the tested biological parameters of the 2nd instar larvae of *Spodoptera frugiperda*

Treatments	LC_25_ (mg L^−1^)	Pupation % ± SD^1^	Pupal duration (days) ± SD	Adult emergency % ± SD	Adult longevity (days) ± SD	Adult fecundity^2^% ± SD	Eggs hatching % ± SD
*Beauveria bassiana* (32 × 10^6^ CFU mg^−1^ WP)	89.67	79.33^b^ ± 3.06	11.33^a^ ± 2.08	89.91^b^ ± 0.39	7.33^a^ ± 1.53	0.00^c^ ± 0.00	-
Spinetoram (12% SC)	0.019	76.33^b^ ± 1.53	14.00^a^ ± 1.00	75.99^c^ ± 0.34	7.00^a^ ± 1.73	19.74^b^ ± 1.99	16.42^b^ ± 15.29
Control	0.00	96.33^a^ ± 2.08	6.00^b^ ± 1.73	95.50^a^ ± 0.58	8.33^a^ ± 1.53	797.90^a^ ± 4.04	72.51^a^ ± 2.12

^1^Standard deviation

^2^Number of eggs/laid female

(-): the treated larvae did not succeed to produce eggs and thus it failed to complete its life cycle

• One-way ANOVA was used to ascertain differences between treatments. Means in each column possess the same letter indicate no significant difference at *P* = 0.05

**Table 3:** Effect of tested insecticides at their LC_25_ values on the tested biological activities of the 4th instar larvae of *Spodoptera frugiperda*

Treatments	LC_25_ (mg L^−1^)	Pupation % ± SD^1^	Pupal duration (days) ± SD	Adult emergency % ± SD	Adult longevity (days) ± SD	Adult fecundity^2^% ± SD	Eggs hatching % ± SD
*Beauveria bassiana* (32 × 10^6^ CFU mg^−1^ WP)	178.67	48.00^c^ ± 14.73	14.67^a^ ± 0.58	91.05^b^ ± 0.40	7.67^a^ ± 1.15	9.88^c^ ± 2.19	0.00^c^ ± 0.00
Spinetoram (12% SC)	0.048	79.67^b^ ± 4.04	14.33^a^ ± 0.58	79.49^c^ ± 0.47	7.33^a^ ± 1.53	52.84^b^ ± 0.83	35.98^b^ ± 7.41
Control	0.00	98.33^a^ ± 2.08	7.33^b^ ± 0.58	96.27^a^ ± 0.61	9.33^a^ ± 0.58	847.66^a^ ± 5.36	76.92^a^ ± 2.87

^1^Standard deviation

^2^Number of eggs/laid female

One-way ANOVA was used to ascertain differences between treatments. Means in each column possess the same letter indicate no significant difference at *P* = 0.05

Likewise, the data of the 4th instar larvae of *S. frugiperda* exhibited significant decreases on the adult emergency when exposed to spinetoram at LC_25_ (79.49%), which was higher than *B. bassiana* at LC_25_ (91.05%) in compare to the control treatment (96.27%). In contrary, significant reduction in the adult fecundity was revealed when exposed to *B. bassiana* at LC_25_ (9.88 eggs) more than LC_25_ of spinetoram (52.84 eggs) compared to the control (847.66 eggs). In addition, significant reduction in the eggs hatching was realized by *B. bassiana* at LC_25_ (0.00%) that exceeded the influence of LC_25_ of spinetoram (35.98%) compared to the control (76.92%) (Table 3).

### Enzymatic activity

#### Lipase activity

**Fig. 1:**
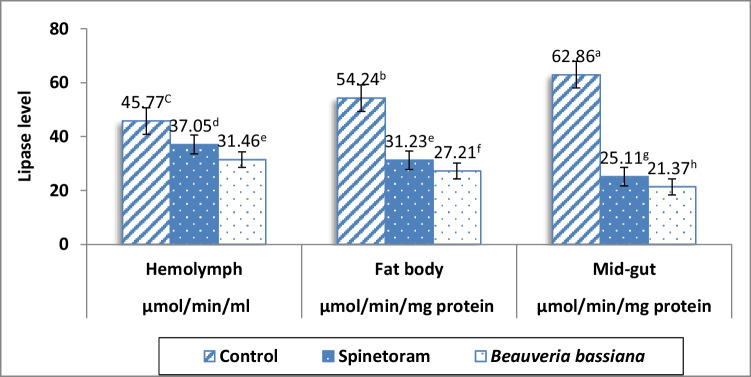
Lipase level in hemolymph, fat bodies, and mid-gut of the 4th instar larvae of *Spodoptera frugiperda* at 48 h of exposure to *Beauveria bassiana* and spinetoram in compare to the control. Means followed by different letters are significantly different at *P* < 0.05

Data of lipase activity in each of hemolymph, fat bodies and mid-gut of the 4th instar larvae of *S. frugiperda* had been significantly decreased whenever exposed to *B. bassiana* more than spinetoram at 48 h and both insecticides showed significant drops more than the control treatment (Fig. 1).

Lipase activity in hemolymph significantly decreased in *B. bassiana* (31.46 μmol/min/mL) more than spinetoram (37.05 μmol/min/mL). Both insecticides showed significant drop more than the control (45.77 μmol/min/mL). Fat bodies showed highest level of lipase in the control treatment that excelled spinetoram and *B. bassiana* with values of 54.24, 31.23, and 27.21 μmol / min / mg protein, respectively. Likewise, mid-gut owned the highest lipase activity in the control that transcend spinetoram and *B. bassiana* with values of 62.86, 25.11, and 21.37 μmol/min/mg protein, respectively (Fig. 1).

#### Glutathione-S-Transferase activity

**Fig. 2:**
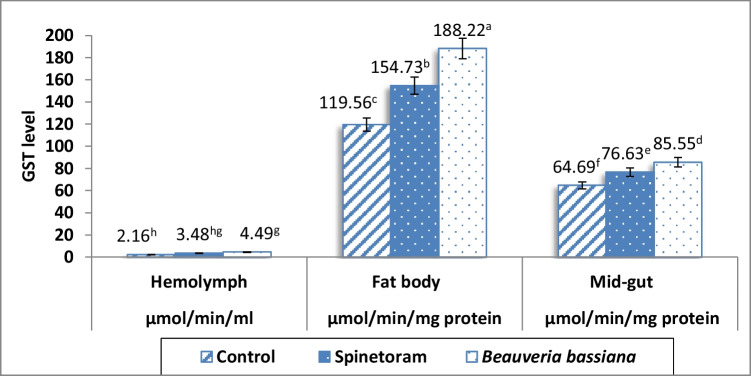
Glutathione-S-transferase level in hemolymph, fat bodies and mid-gut of the 4th instar larvae of *Spodoptera frugiperda* at 48 h of exposure to *Beauveria bassiana* and spinetoram in compare to the control. Means followed by different letters are significantly different at *P* < 0.05

Data of GST level in hemolymph, fat bodies, and mid-gut of the 4th instar larvae of *S. frugiperda* at 48 h of exposure to *B. bassiana* and spinetoram in compare to the control treatment were illustrated in Fig. 2.

Activity of GST in hemolymph for both treatments of *B. bassiana* and spinetoram came with equal level within the values of 4.49 and 3.48 μmol/min/mL, respectively. The GST level in hemolymph in both treatments significantly surpassed its level in the control treatment (2.16 μmol/min/mL).

The fat bodies possessed the highest level of GST in treatment of *B. bassiana* followed by spinetoram and lasted by the control with values of 188.22, 154.73, and 119.56 μmol/min/mg protein, respectively. In the same trend, mid-gut had the highest level of GST in *B. bassiana* followed by spinetoram more than the control with values of 85.55, 76.63, and 64.69 μmol/min/mg protein, respectively (Fig. 2).

#### Residual toxicity

Mortality percentages and residual toxicity of the applied FRs of the tested insecticides against each of the 2nd and 4th instar larvae of *S. frugiperda* at 48 h of exposure were conducted during the growing seasons of maize crop in 2020 and 2021 (Tables 4 and 5).

- Residual toxicity in season 2021

**Table 4:** Mortality percentages in semi-field trials of the 2nd and 4th instar larvae of *Spodoptera frugiperda* (2 G) at 48 h of exposure to the selected insecticides at their applied field rates in season of 2021

Instars	Tested insecticides	Mean of mortality % ± SE^1^ at 48 h of exposure along the 10 DATs					Overall mean of mortality % ± SD^2^
		0 DAT	3 DAT	5 DAT	7 DAT	10 DAT	
2nd	***Beauveria bassiana* (**32 × 10^6^ CFU mg^−1^ WP)	66.67^b^	60.00^b^	56.67^b^	36.67^c^	0.00^e^	44.00^b^
		± 0.58	± 2.00	± 2.08	± 0.58	± 0.00	± 1.05
	**Spinetoram (**12% SC)	96.67^a^	96.67^a^	90.00^ba^	26.67^dc^	20.00^d^	66.00^a^
		± 0.58	± 0.58	± 1.00	± 1.15	± 1.00	± 0.86
4th	***Beauveria bassiana* (**32 × 10^6^ CFU mg^−1^ WP)	60.00^b^	56.67^b^	53.33^cb^	3.33^e^	0.00^e^	34.67^b^
		± 0.00	± 0.58	± 0.58	± 0.58	± 0.00	± 0.55
	**Spinetoram (**12% SC)	93.33^a^	93.33^a^	40.00^c^	20.00^d^	3.33^e^	50.00^a^
		± 0.58	± 0.58	± 2.00	± 2.00	± 0.58	± 1.15

^1^Standard error

^2^Standard deviation

• For each instar separately, mortality percentages with the same letter are not significantly different according to the LSD_0.05_ for the interactions between treatments and intervals of DATs

• For each instar separately, means of overall mortality percentages of the treatments with the same letter are not significantly different according to the LSD_0.05_

The obtained data of the 2nd instar larvae of *S. frugiperda* (Table 4) affirmed that the potent toxic effects attained by *B. bassiana* were 66.67, 60.00, and 56.67% at 0, 3, and 5 DAT, respectively, which were significantly lower than the corresponding potent effects of spinetoram that reached 96.67 and 96.67% at 0 and 3 DAT, respectively. Thereafter, *B. bassiana* showed significant decreases in its toxic effects at 7 DAT and completely vanished at 10 DAT. Meantime, spinetoram also had significant decreases in its toxic effects along the period from 5 to 10 DAT. Ultimately, the result of overall mean of mortality percentage showed that *B. bassiana* (44.00%) was significantly lower than spinetoram (66.00%) on the 2nd instar larvae (Table 4). On the other hand, the data of the 4th instar larvae of *S. frugiperda* (Table 4) corroborated that *B. bassiana* fulfilled its potent toxic effects (60 and 56.67%), which were significantly lower than the corresponding potent effects of spinetoram (93.33%) along the period of 0–3 DAT, respectively. Henceforth, the toxic effects of *B. bassiana* showed significant decreases from 5 up to 7 DAT and then vanished at 10 DAT. Meantime, the toxic effects spinetoram significant decreased from 5 up to 10 DAT. Finally, the results of overall mean of mortality percentages of *B. bassiana* (34.67%) were significantly lower than spinetoram (50.00%) on the 4th instar larvae of *S. frugiperda* (Table 4).

b. Residual toxicity in season 2022

The data of the 2nd instar larvae of *S. frugiperda* (Table 5) affirmed that the potent toxic effects attained by *B. bassiana* (80.00 and 73.33%) were significantly lower than the corresponding effects of spinetoram (100.00 and 96.67%) at 0 and 3 DAT, respectively. Afterwards, treatment of *B. bassiana* showed significant decreases in its toxic effects at 5 DAT and completely vanished at 10 DAT. Likewise, toxic effects of spinetoram significantly decreased along the period from 5 to 10 DAT. Finally, the result of overall mean of mortality percentage showed that *B. bassiana* (43.33%) was significantly lower than spinetoram (73.33%) on the 2nd instar larvae (Table 5). On the other hand, the data of the 4th instar larvae of *S. frugiperda*) (Table 5) demonstrated that spinetoram fulfilled potent toxic effects of 96.67% that significantly surpassed the *B. bassiana* effects of 73.33 and 66.67% along the period of 0–3 DAT, respectively. Henceforth, the toxic effects of *B. bassiana* showed significant decreases at 5 DAT and then vanished at a period from 7 up to 10 DAT, whereas spinetoram significant decreased from 5 up to 10 DAT. Finally, the results of overall mean of mortality percentages of spinetoram (58.67%) significantly excelled *B. bassiana* (34.00%) on the 4th instar larvae of *S. frugiperda*) (Table 5).

**Table 5:** Mortality percentages in semi-field trials of the 2nd and 4th instar larvae of *Spodoptera frugiperda* (2 G) at 48 h of exposure to the selected insecticides at their applied field rates in season of 2022

Instars	Tested insecticides	Mean of mortality % ± SE^1^ at 48 h of exposure along the 10 DATs					Overall mean of mortality % ± SD^2^
		0 DAT	3 DAT	5 DAT	7 DAT	10 DAT	
2nd	***Beauveria bassiana*** (32 × 10^6^ CFU mg^−1^ WP)	80.00^c^	73.33^c^	43.33^e^	20.00^f^	0.00^ g^	43.33^b^
		± 1.00	± 0.58	± 0.58	± 0.00	± 0.00	± 0.43
	**Spinetoram (**12% SC)	100.00^a^	96.67^a^	90.00^b^	56.67^d^	23.33^f^	73.33^a^
		± 0.00	± 0.58	± 1.00	± 0.58	± 0.58	± 0.55
4th	***Beauveria bassiana*** (32 × 10^6^ CFU mg^−1^ WP)	73.33^b^	66.67^b^	30.00^e^	0.00^f^	0.00^f^	34.00^b^
		± 1.15	± 0.58	± 1.00	± 0.00	± 0.00	± 0.55
	**Spinetoram (**12% SC)	96.67^a^	96.67^a^	56.67^c^	43.33^d^	0.00^f^	58.67^a^
		± 0.58	± 0.58	± 0.58	± 0.58	± 0.00	± 0.46

^1^Standard error

^2^Standard deviation

• For each instar separately, mortality percentages with the same letter are not significantly different according to the LSD_0.05_ for the interactions between treatments and intervals of DATs

• For each instar separately, means of overall mortality percentages of the treatments with the same letter are not significantly different according to the LSD_0.05_

## Discussion

Coincidentally, the attempts in the current study were conducted by selecting *Beauveria bassiana* and spinetoram as adequate epitomes for biorational insecticides against *S. frugiperda.* This study was promoted to the Egyptian ministerial decree and adoption for assessments of the most specified and biorational insecticides under the guidance of FAO to set up precautionary implementation to control the new intruder, *S. frugiperda* before entering Egypt on May 30, 2019, and hitherto (IPPC 2019). The importance of biological aspects studies on the *S. frugiperda* realized in knowing how control strategies can be optimized in terms of the appropriate timing and the most effective management measures against this pest in the future that keeps crops away from damage (Rot et al. 2022; Kona et al. 2021). In this respect, the current data of the selected insecticides at their LC_25_ values showed equipollent decreases on pupation percentages and prolongation on the pupal duration of the 2nd instar larvae, likewise the prolongation effect of pupal duration of the 4th instar larvae. On contrary, investigation of Gao et al. (2021) showed that the sub-lethal concentrations of spinetoram had no effect on the pupal duration and pupation percentage of the *S. frugiperda*, while the findings of Nelly et al. (2023) were in agree with our findings that the sub-lethal concentrations of *B. bassiana* could decreased the pupation formation to the minimum rate of the *S. frugiperda*. Our data exhibited significant reductions on the adult emergency percentages on both instar larvae of *S. frugiperda* when exposed to spinetoram more than *B. bassiana* at their LC_25_ values. In contrary, Gao et al. (2021) found that the exposed 3rd instar larvae of *S. frugiperda* to sub-lethal concentrations of spinetoram had no effects on the emergence rate of adults. Nevertheless, feeding of the 3rd to 6th instars larvae of *S. frugiperda* on maize seedlings inoculated with *B. bassiana* (1 × 10^6^ spores mL^−1^) reproduced fewer adult male moths compared to the control treatment (Kuzhuppillymyal-Prabhakarankutty et al. 2021). We also observed no significant changes in the adult longevities of both instar larvae treated with the selected insecticides and their control treatments. These observations were confirmed for spinetoram (Gao et al. 2021), but Sari et al. (2022) found considerable decreases in the female and male adult longevities. Furthermore, the obtained data of laid eggs by the adult female rates were perfectly obstructed by LC_25_ values of *B. bassiana*, much more than spinetoram especially on the 2nd instar larvae that failed to survive for their life cycle completion. The study of Gao et al. (2021) showed no effects on the female fecundity of the exposed 3rd instar larvae of *S. frugiperda* to sub-lethal concentrations of spinetoram. Meanwhile, spinetoram revealed significant reduction on the female fecundity of *Helicoverpa armigera* (Hübner) (Wei et al. 2018). Moreover, our study found that the egg hatchability percentages were diminished by *B. bassiana*, compared to spinetoram. These results meet the deduction of Idrees et al. (2021) who found that EPF isolates of *B. bassiana* could be used as convenient bio-pesticides in the integrated *S. frugiperda* control due to its potent reductions on hatchability of eggs caused by egg mortality percentages in the range of 40 up to 85.6% at different lethal concentrations. On the other hand, Wei et al. (2018) showed that the oral exposure after 24 h of spinetoram at LC_20_ and LC_8_ had great reduction on eggs hatchability of *H. armigera* with 93.19 and 44.77%, respectively.

The enzymes activity of lipase and GST in the current study was selected, as important biochemical parameters, to determine the response of the exposed larvae of *S. frugiperda* to the sub-lethal concentration of the test insecticides. Lipase is essential for many physiological processes even in highly carbohydrates-feeding insects as it facilitates the intake, storage, and mobilization of lipids in their tissues (Walaba et al. 2010; Santana et al. 2017). Meanwhile, GST confers insecticide resistance either via confiscation of the toxic metabolites of insecticides or limiting oxidative stress induced in the exposed insects (Pavlidi et al. 2018). In the present biochemical studies concerning lipase activity in each of hemolymph, fat bodies and mid-gut of the 4th instar larvae of *S. frugiperda* showed significant low level at 48 h by *B. bassiana* in compare to the control, which had the highest lipase activity. This data could be justified by Mondal et al. (2016) who demonstrated that however the culture of the entomopathogenic fungi *B. bassiana* in vitro during the early stationary phase (up to the 5th day post-germination) could not induce lipase production, *B. bassiana* could had partially efficacy in the lack of lipase production. Lipase may be critically needed just at the time of fungal growth within the insect hemocoel that contain a relative high content of lipids just to penetrate the insect integument. Henceforth, lipase role was no longer required (Hegedus and Khachatourians 1988; Zhang et al. 2012). Pathogenic activity of *B. bassiana* mainly attributed to the production of lipase enzyme (Vici et al. 2015). Lipase role the process of hydrolyses on ester bonds of lipoproteins, fats, and waxes to facilitate the penetration of the fungal growth in the interior integument of the insect (Silva et al. 2010; Dhawan and Joshi 2017). On the other hand, no previous reviews had been explained the reason of lipase reduction by spinetoram in each of hemolymph, fat bodies, and mid-gut of the 4th instar larvae of *S. frugiperda*. Furthermore, the present study on GST activity in hemolymph in both treatments significantly surpassed its level in the control. In addition, fat bodies and mid-gut possessed the highest level of GST in the treatment of *B. bassiana* followed by spinetoram and lasted by the control. These data were supported by the detailed analysis of GST achieved by Chen et al. (2022) in *S. frugiperda* participated in the response to the mode of action and detoxification mechanism against spinetoram. Our data were also reinforced by Ahmed et al. (2022) who found significant increases of GST activity at 96 h post-treatment with LC_25_ of spinetoram on the susceptible strains of *S. littoralis*. On the other hand, our obtained data of GST activity by *B. bassiana* treatment came in accordance to the increases of GST activity in *S. litura* as a response to secondary metabolite mycotoxins produced by *B. brongniartii* (Wu et al. 2020). Moreover, Ramzi and Zibaee (2014) hinted that the increases occurred in GST activity could be referred to the influence of some released toxins during the EPF growth on the larval body of *Chilo suppressalis* (Walker) (Lepidoptera: Crambidae). This enzyme liberates phosphoric basis from cuticle of insect integument, which is facility releasing nutrient compounds.

The obtained data throughout the semi-field trials on each of the 2nd and 4th instar larvae of *S. frugiperda* in both successive seasons had potent toxic effects for spinetoram much more than *B. bassiana* at their FRs. These potent effects for both tested insecticides were exhibited during the first 3 DAT. Additionally, the overall mean of mortality percentages showed that spinetoram at FR excelled *B. bassiana* at FR on both instar larvae in both seasons. Therefore, the current study could recommend the field rates of spinetoram and *B. bassiana* as successful biorational insecticides against *S. frugiperda* on maize crops*.* These findings came in accordance with the assertion that spinetoram was the most effective insecticide in controlling *S. frugiperda* along 10 weeks after maize planting versus to emamectin benzoate and chlorantraniliprole (Nonci et al. 2020). On the other hand, the results of *B. bassiana* that possessed the highest toxic action among tested EPF gave rise *B. bassiana* to be effective in controlling *S. frugiperda* larvae in early instars (Shahzad et al. 2021). The field application of *B. bassiana* as a seed treatment in maize could significantly lessen *S. frugiperda* injury and economic damage, and exhibit safeness for beneficial insects (Kuzhuppillymyal-Prabhakarankutty et al. 2021).

## Conclusions

Eventually, our study demonstrated that the larvae of *S. frugiperda* in the early and mid-instars exposed to the sub-lethal concentration of spinetoram could realize significant reductions on their adult emergency compared to *B. bassiana*, whereas rates of fecundity and eggs hatching showed great elimination especially on the early instar larvae exposed to the sub-lethal concentration of *B. bassiana* more than spinetoram. Along 48 h, enzymatic activity of lipase had considerable depression in hemolymph, fat bodies, and mid-gut of the 4th instar larvae in *B. bassiana* more than spinetoram. Both insecticides had the same level of GST in hemolymph, which exceeded the control, while *B. bassiana* possessed the highest GST activity in fat bodies and mid-gut more than spinetoram and lasted by the control. However, the overall mean of mortality percentages in the semi-field trials along two successive seasons showed that spinetoram significantly surpassed *B. bassiana* at their applied FRs on both early and mid-instars larvae; *B. bassiana* could be effectively afford good control on the early instar larvae of *S. frugiperda* particularly during the first 5 DAT.

## Recommendations

Throughout the current study, we could recommend the early intervention by foliar spray applications of the biorational insecticides, spinetoram, and *B. bassiana* to afford a more safe and successful controlling against the early instar larvae of *S. frugiperda *on maize crops.

### Supplementary Information

Below is the link to the electronic supplementary material.

Supplementary file1 (DOCX 83 KB)

Supplementary file2 (DOCX 46 KB)

## Data Availability

At their applied field rates of 50 ml and 400 gm 200 L^-1^ water fadan^-1^, respectively.

## Abbreviations

LS: Lab strain
LC: Lethal concentration
FR: Field rate
hr: Hour
CFU: Colony-forming unit per milliliter mg^−1^
DAT: Day after treatment
*S. frugiperda*: *Spodoptera frugiperda*
*B. bassiana*: *Beauveria bassiana*
GST: Glutathione-S-transferase
EPF: Entomopathogenic fungi
ARS: Agriculture research station

## References

[CR1] Abbott WS (1925) A method for computing the effectiveness of an insecticide. J Econ Entomol 18:265–267 10.1093/jee/18.2.265a

[CR2] Ahmed FS, Helmy YS, Helmy WS (2022) Toxicity and biochemical impact of methoxyfenozide/spinetoram mixture on susceptible and methoxyfenozide-selected strains of *Spodoptera littoralis* (Lepidoptera: Noctuidae). Sci Rep 12:6974. 10.1038/s41598-022-10812-w 35484385 10.1038/s41598-022-10812-w PMC9050723

[CR3] Ali S, Huang Z, Ren SX (2009) Production and extraction of extracellular lipase from the entomopathogenic fungus *Isaria fumosoroseus* (Cordycipitaceae: Hypocreales). Biocont Sci Technol 19:81–89 10.1080/09583150802588524

[CR4] Chen H, Xie M, Lin L, Zhong Y, Zhang F, Su W (2022) Transcriptome analysis of detoxification-related genes in *Spodoptera frugiperda* (Lepidoptera: Noctuidae). J Insect Sci 22(1):1–8. 10.1093/jisesa/ieab108 35134188 10.1093/jisesa/ieab108 PMC8824446

[CR5] Dahi HF, Salem SAR, Gamil WE, Mohamed HO (2020) Heat requirements for the fall armyworm Spodoptera frugiperda (J. E. Smith) (Lepidoptera: Noctuidae) as a new invasive pest in Egypt. Egypt Acad J Biolog Sci 13(4):73–85

[CR6] Day R, Abrahams P, Bateman M, Beale T, Clottey V, Cock M, Colmenarez Y, Corniani N, Early R, Godwin J, Gomez J, Moreno PG, Murphy ST, Oppong-Mensah B, Phiri N, Pratt C, Silvestri S, Witt A (2017) Fall armyworm: impacts and implications for Africa. Outlooks Pest Manag 28(5):196–201(6). 10.1564/v28_oct_02

[CR7] Dhawan M, Joshi N (2017) Enzymatic comparison and mortality of beauveria bassiana against cabbage caterpillar Pieris brassicae LINN. Braz J Microbiol 48(3):522–529 10.1016/j.bjm.2016.08.004 PMC5498455 28262388

[CR8] Dugdale JS (1988) Lepidoptera - annotated catalogue, and keys to family-group taxa. Fauna New Zealand 14:264

[CR9] El-defrawi ME, Toppozada A, Mansour N, Zeid M (1964) Toxicological studies on the Egyptian cotton leafworm, *Prodenia litura*. I. Susceptibility of different larval instars of *P. litura* to insecticides. J Econ Entomol 57:591–593 10.1093/jee/57.4.591

[CR10] El-Sabrout A (2009) Different effects of some materials from plant origin on the cotton leafworm. M Sc thesis, Alexandria Univ Fac Agri

[CR11] Environmental Protection Agency (2009) Spinetoram technical insecticide. Washington, D.C. 20460 Amend 11 Aug 2009

[CR12] European and Mediterranean Plant Protection Organization (2015) PM 7/124 (1) *Spodoptera littoralis*, *Spodoptera litura*, *Spodoptera frugiperda* and *Spodoptera eridania*. OEPP/EPPO Bull 45(3):410–444 10.1111/epp.12258

[CR13] Finney DJ (1971) Probit analysis, 3rd ed. Cambridge University Press, Cambridge, London, UK pp. 1–333

[CR14] Food and Agriculture Organization (FAO) (2018) Integrated management of the fall armyworm on maize: A guide for farmer field schools in Africa. Rome, Italy p.140 https://www.fao.org/publications

[CR15] Food and Agriculture Organization (FAO) (2019) Report of first detection of Spodoptera frugiperda - Fall Armyworm (FAW) in Egypt. IPPC, Rome Preliminary Report No. EGY-01/1

[CR16] Food and Agriculture Organization (FAO) (2020) The Global Action for fall armyworm Control: Action framework 2020–2022*.* Working together to tame the global threat –Rome. 10.4060/ca9252en

[CR17] Gamil WE (2020) Fall armyworm, Spodoptera frugiperda (J. E. Smith) (Lepidoptera: Noctuidae) biological aspects as a new alien invasive pest in Upper Egypt, Egypt. Acad J Biolog Sci (A. Entomology) 13(3):189–196

[CR18] Gao Z, Chen Y, He K, Guo J, Wang Z (2021) Sub-lethal effects of the microbial derived insecticide spinetoram on the growth and fecundity of the fall armyworm (Lepidoptera: Noctuidae). J Econ Entomol 114(4):1582–1587 34166511 10.1093/jee/toab123

[CR19] García-Munguía AM, Garza-Hernández JA, Rebollar-Tellez EA (2011) Transmission of *Beauveria bassiana* from male to female *Aedes aegypti* mosquitoes. Parasit Vectors 4:24. 10.1186/1756-3305-4-24 21352560 10.1186/1756-3305-4-24 PMC3051917

[CR20] Goergen G, Kumar PL, Sankung SB, Togola A, Tamò M (2016) First report of outbreaks of the fall armyworm *Spodoptera frugiperda* (J E Smith) (Lepidoptera, Noctuidae), a new alien invasive pest in West and Central Africa. PLOS ONE 11(10):e0165632. 10.1371/journal.pone.0165632 27788251 10.1371/journal.pone.0165632 PMC5082806

[CR21] Hailu G, Niassy S, Bässler T, Ochatum N, Studer C, Salifu D, Agbodzavu MK, Khan ZR, Midega C, Subramanian S (2021) Could fall armyworm, *Spodoptera frugiperda* (J. E. Smith) invasion in Africa contribute to the displacement of cereal stem borers in maize and sorghum cropping systems. Inter J Tropical Insect Sci 41:1753–1762 10.1007/s42690-020-00381-8

[CR22] Hegedus DD, Khachatourians GG (1988) Production of an extracellular lipase by Beauveria bassiana. Biotechnol Lett 10:637–642. 10.1007/BF01024716

[CR23] Idrees A, Qadir ZA, Akutse KS, Afzal A, Hussain M, Islam W, Waqas MS, Bamisile BS, Li J (2021) Effectiveness of entomopathogenic fungi on immature stages and feeding performance of fall armyworm, *Spodoptera frugiperda* (Lepidoptera: Noctuidae) larvae. Insects 12:1044. 10.3390/insects12111044 34821844 10.3390/insects12111044 PMC8624455

[CR24] Insecticides Resistance Action Committee (2020) IRAC mode of action classification scheme. Approved version 9.4. Croplife Inter 1–30. https://www.irac-online.org

[CR25] International Plant Biotechnology Outreach (2019) Maize in Africa. IPBO p.55. https://ipbo.vib-ugent.be/wp-content/uploads/2015/02/vib_fact_MaizeForAfrica_EN_2017_LR.pdf

[CR26] Ismail SM (2020) Effect of sub-lethal doses of some insecticides and their role on detoxication enzymes and protein-content of Spodoptera littoralis (Boisd.) (Lepidoptera: Noctuidae). Bull Natl Res Cent 44:35 10.1186/s42269-020-00294-z

[CR27] Kassie M, Wossen T, Groote HD, Tefera T, Sevgan S, Balew S (2020) Economic impacts of fall armyworm and its management strategies: evidence from Southern Ethiopia. Eur Rev Agric Econ 47(4):1473–1501. 10.1093/erae/jbz048 10.1093/erae/jbz048

[CR28] Kona NEM, Taha AK, Mahmoud MEE, Adam AHM (2021) The biology of Fall armyworm (*Spodoptera frugiperda*. J. E. Smith) in Sudan. J Agron Res 4(1):1–5. 10.14302/issn.2639-3166.jar-21-3858 10.14302/issn.2639-3166.jar-21-3858

[CR29] Kuzhuppillymyal-Prabhakarankutty L, Ferrara-Rivero FH, Tamez-Guerra P, Gomez-Flores R, Rodríguez-Padilla MC, Ek-Ramos MJ (2021) Effect of *Beauveria bassiana* seed treatment on *Zea mays* L. response against *Spodoptera frugiperda*. Appl Sci 11:2887. 10.3390/app11072887 10.3390/app11072887

[CR30] Li X, Schuler MA, Berenbaum MR (2007) Molecular mechanisms of metabolic resistance to synthetic and natural xenobiotics. Annu Rev Entomol 52:231–253. 10.1146/annurev.ento.51.110104.151104 16925478 10.1146/annurev.ento.51.110104.151104

[CR31] Lumjuan N, Mc Carroll L, Prapanthadara L, Hemingway J, Ranson H (2005) Elevated activity of an epsilon class glutathione transferase confers DDT resistance in the dengue vector, *Aedes aegypti*. Insect Biochem Mol Biol 35:861–871. 10.1016/j.ibmb.2005.03.008 15944082 10.1016/j.ibmb.2005.03.008

[CR32] Martin R, Montgomery S, Phan S, Im S (2016) Maize production guide for Cambodian conditions. Australian Centre for International Agricultural Research (ACIAR), Canberra, Monograph No. 167, pp. 84.

[CR33] Mohamed HO, El-Heneidy AH, Dahi HF, Awad AA (2022) First record of the fall armyworm, *Spodoptera frugiperda* (J. E. Smith) (Lepidoptera: Noctuidae) on sorghum plants, a new invasive pest in Upper Egypt, Egypt. Acad J Biolog Sci (A. Entomol) 15(1):15–23

[CR34] Mondal S, Baksi S, Koris A, Vatai G (2016) Journey of enzymes in entomopathogenic fungi. Pac Sci Rev A: Nat Sci Eng 18:85e99

[CR35] Montezano DG (2018) Host plants of *Spodoptera frugiperda* (Lepidoptera: Noctuidae) in the Americas. Afr Entomol 26:286–300 10.4001/003.026.0286

[CR36] Moore TC (2018) Counting cells with hemocytometer. Protocols Io. 10.17504/protocols.io.nxsdfne 10.17504/protocols.io.nxsdfne

[CR37] Mwamburi LA (2021) Endophytic fungi, *Beauveria bassiana* and *Metarhizium anisopliae*, confer control of the fall armyworm, *Spodoptera frugiperda* (J. E. Smith) (Lepidoptera: Noctuidae), in two tomato varieties. Egy J Biol Pest Control 31:7. 10.1186/s41938-020-00357-3 10.1186/s41938-020-00357-3

[CR38] Nelly N, Reflinaldon, Meriqorina SR (2023) Effective concentration of entomopathogens Beauveria bassiana (Bals) Vuil as biological control agents for Spodoptera frugiperda J.E. Smith (Lepidoptera: Noctuidae). IOP Conf Ser Ear Environ Sci 1160(1):012035 10.1088/1755-1315/1160/1/012035

[CR39] Nonci N, Pakki S, Muis A (2020) Field testing of synthetic insecticides on fall armyworm, *Spodoptera frugiferda* (J.E. Smith) in corn plant. Earth Environ Sci 911:012059

[CR40] Pavlidi N, Vontas J, Leeuwen TV (2018) The role of glutathione S-transferases (GSTs) in insecticide resistance in crop pests and disease vectors. Curr Opin Insect Sci 27:97–102. 10.1016/j.cois.2018.04.007 30025642 10.1016/j.cois.2018.04.007

[CR41] Ramos Y, Taibo AD, Jiménez JA (2020) Endophytic establishment of *Beauveria bassiana* and *Metarhizium anisopliae* in maize plants and its effect against *Spodoptera frugiperda* (J. E. Smith) (Lepidoptera: Noctuidae) larvae. Egypt J Biol Pest Control 30:20. 10.1186/s41938-020-00223-2 10.1186/s41938-020-00223-2

[CR42] Ramzi S, Zibaee A (2014) Biochemical properties of different entomopathogenic fungi and their virulence against *Chilo suppressalis* (Lepidoptera: Crambidae) larvae. Biocont Scie Technol 24(5):597–610. 10.1080/09583157.2014.883360 10.1080/09583157.2014.883360

[CR43] Rot M, Maistrello L, Costi E, Trdan S (2022) Biological parameters, phenology and temperature requirements of *Halyomorpha halys* (Hemiptera: Pentatomidae) in the Sub-Mediterranean climate of Western Slovenia. Insects 13:956. 10.3390/insects13100956 36292903 10.3390/insects13100956 PMC9604413

[CR44] Santana CC, Barbosa LA, Júnior IDB, Do Nascimento TG, Dornelas CB, Grillo LAM (2017) Lipase activity in the larval midgut of *Rhynchophorus palmarum*: biochemical characterization and the effects of reducing agents. Insects 8:100. 10.3390/insects8030100 28902170 10.3390/insects8030100 PMC5620720

[CR45] Sari JMP, Herlinda S, Suwandi S (2022) Endophytic fungi from South Sumatra (Indonesia) in seed-treated corn seedlings affecting development of the fall armyworm, Spodoptera frugiperda J.E. Smith (Lepidoptera: Noctuidae). Egy J Biol Pest Cont 32:103 10.1186/s41938-022-00605-8

[CR46] Shah PA, Pell JK (2003) Entomopathogenic fungi as biological control agents. Appl Microbiol Biotechnol 61:413–423 12764556 10.1007/s00253-003-1240-8

[CR47] Shahzad MA, Irfan M, Wahab AA, Zafar F, Abdulrehman (2021) Toxicity of entomopathogenic fungi against *Spodoptera frugiperda* larvae under laboratory conditions. J Agric Sc Food Technol 7(3):355–358. 10.17352/2455-815X.000131 10.17352/2455-815X.000131

[CR48] Silva WOB, Santi L, Schrank A, Vainstein MH (2010) *Metarhizium anisopliae* lipolytic activity plays a pivotal role in *Rhipicephalus* (*Boophilus*) *microplus* infection. Fungal Biol 114:10–15 20965056 10.1016/j.mycres.2009.08.003

[CR49] Sinha KK, Choudhary AK, Kumari P (2016) Entomopathogenic fungi. In: Vici AC, da Cruz AF, Facchini FD, de Carvalho CC, Pereira MG, Fonseca-Maldonado R, Ward RJ, Pessela BC, Fernandez-Lorente G, Torres FA, Jorge JA, Polizeli ML (ed) Beauveria bassiana Lipase A expressed in Komagataella (Pichia) pastoris with potential for biodiesel catalysis. Front Microbiol 6: 1083. 10.3389/fmicb.2015.01083 10.3389/fmicb.2015.01083 PMC4595793 26500628

[CR50] Statistical Analysis System (SAS) Institute (2002). PC-SAS user guide, version 8, 6th Edition, North Carolina Statistical Analysis System Institute, Inc.

[CR51] Supakdamrongkul P, Bhumiratana A, Wiwat C (2010) Characterization of an extracellular lipase from the biocontrol fungus, Nomuraea rileyi MJ, and its toxicity toward Spodoptera litura. J Invertebr Pathol 105:228–235 10.1016/j.jip.2010.06.011 20600093

[CR52] Timilsena BP, Niassy S, Kimathi E, Abdel-Rahman EM, Seidl-Adams I, Wamalwa M, Tonnang HEZ, Ekesi S, Hughes DP, Rajotte EG, Subramanian S (2022) Potential distribution of fall armyworm in Africa and beyond, considering climate change and irrigation patterns. Sci Rep 12:539 35017586 10.1038/s41598-021-04369-3 PMC8752590

[CR53] Vici AC, da Cruz AF, Facchini FDA, de Carvalho CC, Pereira MG, Fonseca-Maldonado R, Ward RJ, Pessela BC, Fernandez-Lorente G, Torres FAG, Jorge JA, Polizeli MLTM (2015) Beauveria bassiana Lipase A expressed in Komagataella (Pichia) pastoris with potential for biodiesel catalysis. Front Microbiol 6:1083 10.3389/fmicb.2015.01083 PMC4595793 26500628

[CR54] Walaba DL, Hoffmann KH, Woodring J (2010) Control of the release of digestive enzymes in the larvae of the fall armyworm, *Spodoptera Frugiperda*. Arch Insect Biochem Physiol 73(1):14–29 19771560 10.1002/arch.20332

[CR55] Wei J, Zhang L, Yang S, Xie B, An S, Liang G (2018) Assessment of the lethal and sub-lethal effects by spinetoram on cotton bollworm. PLOS ONE 13(9):e0204154. 10.1371/journal.pone.0204154 30216388 10.1371/journal.pone.0204154 PMC6138415

[CR56] Wu J, Li J, Zhang C, Yu X, Cuthbertson AGS, Ali S (2020) Biological impact and enzyme activities of *Spodoptera litura* (Lepidoptera: Noctuidae) in response to synergistic action of *Matrine* and *Beauveria brongniartii*. Front Physiol 11:584405. 10.3389/fphys.2020.584405 33224038 10.3389/fphys.2020.584405 PMC7667252

[CR57] Zhang S, Wideman E, Bernard G, Lesot A, Pinot E, Pedrini N, Keyhani NO (2012) CYP52X1, representing new cytochrome P450 subfamily, displays fatty acid hydroxylase activity and contributes to virulence and growth on insect cuticular substrates in entomopathogenic fungus *Beauveria bassiana*. J Biol Chem 28:13477e13486 10.1074/jbc.M111.338947 PMC3339963 22393051

