# Correction: Field Efficacy, Sub-lethal, and Biochemical Effects of Certain Biorational Insecticides Against the New Intruder, *Spodoptera frugiperda* in Bani-Suef, Upper Egypt

**DOI:** 10.1007/s13744-024-01199-6

**Published:** 2024-08-14

**Authors:** Wael M. Khamis, Ahmed M. El-Sabrout, Rima Shahin, Elham F. Abdel-Rahim

**Affiliations:** 1 Plant Protection Research Institute, Agricultural Research Center, Al-Sabhia, Alexandria, Egypt; 2 Department of Applied Entomology and Zoology, Faculty of Agriculture (El-Shatby), Alexandria University, Alexandria, 21545 Egypt; 3 Plant Protection Research Institute, Agricultural Research Center, Sides Agriculture Research Station, Al-Giza, Egypt

**Correction: Neotropical Entomology (2023) 52:963–973**

10.1007/s13744-023-01064-y

In the original publication, the first and third affiliation erroneously stated "Agriculture Research Center", while the correct display is: "Agricultural Research Center".

The original article has been corrected and the proper display of the affiliations is also provided here.

