# Supplementary material for: Field Efficacy, Sub-lethal, and Biochemical Effects of Certain Biorational Insecticides Against the New Intruder, Spodoptera frugiperda in Bani-Suef, Upper Egypt
Source: Neotrop Entomol. 2023 Jul 25;52(5):963–73. doi: 10.1007/s13744-023-01064-y (PMC10545592; doi:10.1007/s13744-023-01064-y)
Supplement: Supplementary file 1 — Supplementary file1 (DOCX 83 KB) [file 13744_2023_1064_MOESM1_ESM.docx]

**Experimental Design of Toxicity Test**

|  |  |  | **Treatments** | **Sub-lethal concentrations**  **[Numbers] (ranges)** | | **Leaf discs**  **dipping technique** | |  | **Exposure on ten larvae at 48 hrs** | **Mortality% at 48 hrs** | **Estimated LC_25_** |
| --- | --- | --- | --- | --- | --- | --- | --- | --- | --- | --- | --- |
|  |  |  |  |  |  |  | Castor Leaf discs | → | Cup: 2^nd^ larvae | Abbott’s equation (1925) | Probit analysis Finney (1971) |
|  |  |  |  | [7]  (0.3 x10^7^up to  3 x10^9^  conidia ml^-1^) | Each conc. |  |  |  |  |  |  |
|  |  |  | ***B.bassiana*** |  |  |  | Castor Leaf discs | → | Cup: 2^nd^ larvae |  |  |
|  |  |  |  |  |  |  |  |  |  |  |  |
|  |  |  |  |  |  |  | Castor Leaf discs | → | Cup: 2^nd^ larvae |  |  |
|  |  |  |  |  |  |  |  |  |  |  | Probit analysis Finney (1971) |
|  |  |  |  |  |  |  | Castor Leaf discs | → | Cup: 2^nd^ larvae | Abbott’s equation (1925 |  |
|  |  |  |  | [7]  (0.003 up to 0.50 mg L^-1^) | Each conc. |  |  |  |  |  |  |
|  |  |  | **Spinetoram** |  |  |  | Castor Leaf discs | → | Cup: 2^nd^ larvae |  |  |
|  |  |  |  |  |  |  |  |  |  |  |  |
|  |  |  |  |  |  |  | Castor Leaf discs | → | Cup: 2^nd^ larvae |  |  |
|  |  |  |  |  |  |  |  |  |  |  |  |
|  |  |  |  |  |  |  | Castor Leaf discs | → | Cup: 2^nd^ larvae | Abbott’s equation (1925 | Probit analysis Finney (1971) |
|  |  |  |  | Distilled water |  |  |  |  |  |  |  |
|  |  |  | **Control** |  | - |  | Castor Leaf discs | → | Cup: 2^nd^ larvae |  |  |
|  |  |  |  |  |  |  |  |  |  |  |  |
|  |  |  |  |  |  |  | Castor Leaf discs | → | Cup: 2^nd^ larvae |  |  |
|  |  |  |  |  |  |  |  |  |  |  |  |
| **Toxicity test** |  |  |  | [7]  (0.7 x10^6^ up to  5.8x10^7^  conidia ml^-1^) |  |  | Castor Leaf discs | → | Cup: 4^th^ larvae | Abbott’s equation (1925 |  |
|  |  |  |  |  | Each conc. |  |  |  |  |  | Probit analysis Finney (1971) |
|  |  |  | ***B.bassiana*** |  |  |  | Castor Leaf discs | → | Cup: 4^th^ larvae |  |  |
|  |  |  |  |  |  |  |  |  |  |  |  |
|  |  |  |  |  |  |  | Castor Leaf discs | → | Cup: 4^th^ larvae |  |  |
|  |  |  |  |  |  |  |  |  |  |  |  |
|  |  |  |  | [8]  (2 up to 0.2 mg L^-1^) |  |  | Castor Leaf discs | → | Cup: 4^th^ larvae | Abbott’s equation (1925 |  |
|  |  |  |  |  | Each conc. |  |  |  |  |  | Probit analysis Finney (1971) |
|  |  |  | **Spinetoram** |  |  |  | Castor Leaf discs | → | Cup: 4^th^ larvae |  |  |
|  |  |  |  |  |  |  |  |  |  |  |  |
|  |  |  |  |  |  |  | Castor Leaf discs | → | Cup: 4^th^ larvae |  |  |
|  |  |  |  |  |  |  |  |  |  |  | Probit analysis Finney (1971) |
|  |  |  |  | Distilled water |  |  | Castor Leaf discs | → | Cup: 4^th^ larvae | Abbott’s equation (1925 |  |
|  |  |  |  |  |  |  |  |  |  |  |  |
|  |  |  | **Control** |  | - |  | Castor Leaf discs | → | Cup: 4^th^ larvae |  |  |
|  |  |  |  |  |  |  |  |  |  |  |  |
|  |  |  |  |  |  |  | Castor Leaf discs | → | Cup: 4^th^ larvae |  |  |
|  |  |  |  |  |  |  |  |  |  |  |  |

**Experimental Design of Sub-Lethal Effect**

|  |  |  | **Treatments** | **Dipping technique** | |  | **Exposure test on**  **100 larvae at 48 hrs** | **Biological aspects** | |
| --- | --- | --- | --- | --- | --- | --- | --- | --- | --- |
|  |  |  |  |  | Treated Castor Leaf discs | → | Container (2^nd^ instar larvae) | survival | %Pupation  Pupal duration  %Adult emergence  %Adult fecundity  %Eggs hatching |
|  |  |  |  |  |  |  |  |  |  |
|  |  |  | *B.bassiana* LC_25_ |  | Treated Castor Leaf discs | → | Container (2^nd^ instar larvae) | survival |  |
|  |  |  |  |  |  |  |  |  |  |
|  |  |  | on 2^nd^ instar larvae |  | Treated Castor Leaf discs | → | Container (2^nd^ instar larvae) | survival |  |
|  |  |  |  |  |  |  |  |  |  |
|  |  |  |  |  | Treated Castor Leaf discs | → | Container (2^nd^ instar larvae) | survival | %Pupation  Pupal duration  %Adult emergence  %Adult fecundity  %Eggs hatching |
|  |  |  |  |  |  |  |  |  |  |
|  |  |  | Spinetoram LC_25_ |  | Treated Castor Leaf discs | → | Container (2^nd^ instar larvae) | survival |  |
|  |  |  |  |  |  |  |  |  |  |
|  |  |  | on 2^nd^ instar larvae |  | Treated Castor Leaf discs | → | Container (2^nd^ instar larvae) | survival |  |
|  |  |  |  |  |  |  |  |  |  |
|  |  |  |  |  | Treated Castor Leaf discs with distilled water | → | Container (2^nd^ instar larvae) | survival | %Pupation  Pupal duration  %Adult emergence  %Adult fecundity  %Eggs hatching |
|  |  |  |  |  |  |  |  |  |  |
|  |  |  | Control |  | Treated Castor Leaf discs with distilled water | → | Container (2^nd^ instar larvae) | survival |  |
|  |  |  |  |  |  |  |  |  |  |
|  |  |  |  |  | Treated Castor Leaf discs with distilled water | → | Container (2^nd^ instar larvae) | survival |  |
|  |  |  |  |  |  |  |  |  |  |
| **Sub-lethal**  **effect** |  |  |  |  | Treated Castor Leaf discs | → | Container (4^th^ instar larvae) | survival | %Pupation  Pupal duration  %Adult emergence  %Adult fecundity  %Eggs hatching |
|  |  |  |  |  |  |  |  |  |  |
|  |  |  | *B.bassiana* LC_25_ |  | Treated Castor Leaf discs | → | Container (4^th^ instar larvae) | survival |  |
|  |  |  |  |  |  |  |  |  |  |
|  |  |  | on 4^th^ instar larvae |  | Treated Castor Leaf discs | → | Container (4^th^ instar larvae) | survival |  |
|  |  |  |  |  |  |  |  |  |  |
|  |  |  |  |  | Treated Castor Leaf discs | → | Container (4^th^ instar larvae) | survival | %Pupation  Pupal duration  %Adult emergence  %Adult fecundity  %Eggs hatching |
|  |  |  |  |  |  |  |  |  |  |
|  |  |  | Spinetoram LC_25_ |  | Treated Castor Leaf discs | → | Container (4^th^ instar larvae) | survival |  |
|  |  |  |  |  |  |  |  |  |  |
|  |  |  | on 4^th^ instar larvae |  | Treated Castor Leaf discs | → | Container (4^th^ instar larvae) | survival |  |
|  |  |  |  |  |  |  |  |  |  |
|  |  |  |  |  | Treated Castor Leaf discs with distilled water | → | Container (4^th^ instar larvae) | survival | %Pupation  Pupal duration  %Adult emergence  %Adult fecundity  %Eggs hatching |
|  |  |  |  |  |  |  |  |  |  |
|  |  |  | Control |  | Treated Castor Leaf discs with distilled water | → | Container (4^th^ instar larvae) | survival |  |
|  |  |  |  |  |  |  |  |  |  |
|  |  |  |  |  | Treated Castor Leaf discs with distilled water | → | Container (4^th^ instar larvae) | survival |  |
|  |  |  |  |  |  |  |  |  |  |

**Experimental Design of Residual Toxicity in Semi-Field Trials**

| **Season** | **Field experiment design of foliar spray application on maize crop** | | | | | | | | |  | **Laboratory toxicity tests under conditions of 27 ±2 °C, RH 60 ± 5%** | | | | |  |
| --- | --- | --- | --- | --- | --- | --- | --- | --- | --- | --- | --- | --- | --- | --- | --- | --- |
|  | **Treatments** | **Field rate**  **(200 L^-1^ water**  **fadan^-1^)** | **Field replicates** | | **RCBD**  **in 360 m^2^** | | | | **Sampling** |  | **instar larvae** | **Rep.** | **Glass cup (250cm^3^)** | **Mortality%**  **at 48 hrs** | **long-term toxicities along DATs** |  |
|  |  |  |  |  | |  |  |  |  |  |  |  |  |  |  |  |
| **First year** |  |  |  | Plot (40 m^2^) | |  |  |  | 10 mid-aged leaves/plot  in perforated bag  at 0,3, 5, 7 and 10 DAT |  | **2^nd^** | 1 | 10 larvae + treated leaves | Abbott (1925). | Overall mean of mortality% |  |
|  |  | 400 gm |  |  |  |  |  |  |  | → |  | 2 | 10 larvae + treated leaves |  |  |  |
|  | ***B.bassiana*** |  |  | Plot (40 m^2^) | |  |  |  |  |  |  | 3 | 10 larvae + treated leaves |  |  |  |
|  |  |  |  |  |  |  |  |  |  |  | **4^th^** | 1 | 10 larvae + treated leaves | Abbott (1925). | Overall mean of mortality% |  |
|  |  |  |  | Plot (40 m^2^) | |  |  |  |  | → |  | 2 | 10 larvae + treated leaves |  |  |  |
|  |  |  |  |  |  |  |  |  |  |  |  | 3 | 10 larvae + treated leaves |  |  |  |
|  |  |  |  |  | |  | | |  |  |  |  |  |  |  | |
|  |  |  |  | Plot (40 m^2^) | |  |  |  | 10 mid-aged leaves/plot  in perforated bag  at 0,3, 5, 7 and 10 DAT |  | **2^nd^** | 1 | 10 larvae + treated leaves | Abbott (1925). | Overall mean of mortality% | |
|  |  | 50 ml |  |  |  |  |  |  |  | → |  | 2 | 10 larvae + treated leaves |  |  |  |
|  | **Spinetoram** |  |  | Plot (40 m^2^) | |  |  |  |  |  |  | 3 | 10 larvae + treated leaves |  |  |  |
|  |  |  |  |  |  |  |  |  |  |  | **4^th^** | 1 | 10 larvae + treated leaves | Abbott (1925). | Overall mean of mortality% | |
|  |  |  |  | Plot (40 m^2^) | |  |  |  |  | → |  | 2 | 10 larvae + treated leaves |  |  |  |
|  |  |  |  |  |  |  |  |  |  |  |  | 1 | 10 larvae + treated leaves |  |  |  |
|  |  |  |  |  | |  | | |  |  |  |  |  |  |  | |
|  |  |  |  | Plot (40 m^2^) | |  |  |  | 10 mid-aged leaves/plot  in perforated bag  at 0,3, 5, 7 and 10 DAT |  | **2^nd^** | 1 | 10 larvae + treated leaves | Abbott (1925). | Overall mean of mortality% | |
|  |  |  |  |  |  |  |  |  |  | → |  | 2 | 10 larvae + treated leaves |  |  |  |
|  | **Control** | water |  | Plot (40 m^2^) | |  |  |  |  |  |  | 3 | 10 larvae + treated leaves |  |  |  |
|  |  |  |  |  |  |  |  |  |  |  | **4^th^** | 1 | 10 larvae + treated leaves | Abbott (1925). | Overall mean of mortality% | |
|  |  |  |  | Plot (40 m^2^) | |  |  |  |  | → |  | 2 | 10 larvae + treated leaves |  |  |  |
|  |  |  |  |  |  |  |  |  |  |  |  | 3 | 10 larvae + treated leaves |  |  |  |
|  |  |  |  |  | |  |  |  |  |  |  |  |  |  |  |  |
|  |  |  |  |  | |  |  |  |  |  |  |  |  |  |  |  |
| **Second year** |  |  |  | Plot (40 m^2^) | |  |  |  | 10 mid-aged leaves/plot  in perforated bag  at 0,3, 5, 7 and 10 DAT |  | **2^nd^** | 1 | 10 larvae + treated leaves | Abbott (1925). | Overall mean of mortality% |  |
|  |  | 400 gm |  |  |  |  |  |  |  | → |  | 2 | 10 larvae + treated leaves |  |  |  |
|  | ***B.bassiana*** |  |  | Plot (40 m^2^) | |  |  |  |  |  |  | 3 | 10 larvae + treated leaves |  |  |  |
|  |  |  |  |  |  |  |  |  |  |  | **4^th^** | 1 | 10 larvae + treated leaves | Abbott (1925). | Overall mean of mortality% |  |
|  |  |  |  | Plot (40 m^2^) | |  |  |  |  | → |  | 2 | 10 larvae + treated leaves |  |  |  |
|  |  |  |  |  |  |  |  |  |  |  |  | 3 | 10 larvae + treated leaves |  |  |  |
|  |  |  |  |  | |  | | |  |  |  |  |  |  |  | |
|  |  |  |  | Plot (40 m^2^) | |  |  |  | 10 mid-aged leaves/plot  in perforated bag  at 0,3, 5, 7 and 10 DAT |  | **2^nd^** | 1 | 10 larvae + treated leaves | Abbott (1925). | Overall mean of mortality% | |
|  |  | 50 ml |  |  |  |  |  |  |  | → |  | 2 | 10 larvae + treated leaves |  |  |  |
|  | **Spinetoram** |  |  | Plot (40 m^2^) | |  |  |  |  |  |  | 3 | 10 larvae + treated leaves |  |  |  |
|  |  |  |  |  |  |  |  |  |  |  | **4^th^** | 1 | 10 larvae + treated leaves | Abbott (1925). | Overall mean of mortality% | |
|  |  |  |  | Plot (40 m^2^) | |  |  |  |  | → |  | 2 | 10 larvae + treated leaves |  |  |  |
|  |  |  |  |  |  |  |  |  |  |  |  | 1 | 10 larvae + treated leaves |  |  |  |
|  |  |  |  |  | |  | | |  |  |  |  |  |  |  | |
|  |  |  |  | Plot (40 m^2^) | |  |  |  | 10 mid-aged leaves/plot  in perforated bag  at 0,3, 5, 7 and 10 DAT |  | **2^nd^** | 1 | 10 larvae + treated leaves | Abbott (1925). | Overall mean of mortality% | |
|  |  |  |  |  |  |  |  |  |  | → |  | 2 | 10 larvae + treated leaves |  |  |  |
|  | **Control** | water |  | Plot (40 m^2^) | |  |  |  |  |  |  | 3 | 10 larvae + treated leaves |  |  |  |
|  |  |  |  |  |  |  |  |  |  |  | **4^th^** | 1 | 10 larvae + treated leaves | Abbott (1925). | Overall mean of mortality% | |
|  |  |  |  | Plot (40 m^2^) | |  |  |  |  | → |  | 2 | 10 larvae + treated leaves |  |  |  |
|  |  |  |  |  |  |  |  |  |  |  |  | 3 | 10 larvae + treated leaves |  |  |  |
