# Supplementary material for: Field Efficacy, Sub-lethal, and Biochemical Effects of Certain Biorational Insecticides Against the New Intruder, Spodoptera frugiperda in Bani-Suef, Upper Egypt
Source: Neotrop Entomol. 2023 Jul 25;52(5):963–73. doi: 10.1007/s13744-023-01064-y (PMC10545592; doi:10.1007/s13744-023-01064-y)
Supplement: Supplementary file 2 — Supplementary file2 (DOCX 46 KB) [file 13744_2023_1064_MOESM2_ESM.docx]

**Table 1** Field survey on *Spodoptera frugiperda* infestation on their hosted crops in some regions of Bani-Suef governorate during seasons of 2021and 2022

| **Regions** | **Villages** | **GPS Site**  **(Latitude : Longitude)** | **Hosted plant** | **Varieties** |
| --- | --- | --- | --- | --- |
| Biba | Ezbit Al-Hakem | 28°54'29.4"N : 30°55'21.9"E | *Zea mays* (L.) | Giza 2 |
|  | ARS, Sids | 28°54'29.5''N : 30°57'01.1"E | *Zea mays* (L.) | Sids128 |
|  |  |  | *Sorghum bicolor* (L.) | Various Hybrids |
| Sumasta | Bani-Hallah | 28^o^54'23.7''N : 30^o^55'08.7"E | *Zea mays* (L.) | Hybrid 2031 |
|  | Ghaftan | 28°57'05.5''N : 30°49'10.3"E | *Sorghum bicolor* (L.) | Baladi |

**Fig. 1** Lethal concentration curve of *Beauveria bassiana* against the 2^nd^ instar larvae of *Spodotera frugiperda* at 48 hrs of exposure.

**Fig. 2** Lethal concentration curve of *Beauveria bassiana* against the 4^th^ instar larvae of *Spodotera frugiperda* at 48 hrs of exposure.

**Fig. 3** Lethal concentration curve of spinetoram against the 2^nd^ instar larvae of *Spodotera frugiperda* at 48 hrs of exposure.

**Fig. 4** Lethal concentration curve of spinetoram against the 4^th^ instar larvae of *Spodotera frugiperda* at 48 hrs of exposure.
